# Effect of MWCNTs Functionalization on Thermal, Electrical, and Ammonia-Sensing Properties of MWCNTs/PMMA and PHB/MWCNTs/PMMA Thin Films Nanocomposites

**DOI:** 10.3390/nano11102625

**Published:** 2021-10-06

**Authors:** Raina Aman Qazi, Rozina Khattak, Luqman Ali Shah, Rizwan Ullah, Muhammad Sufaid Khan, Muhammad Sadiq, Mahmoud M. Hessien, Zeinhom M. El-Bahy

**Affiliations:** 1 National Centre of Excellence in Physical Chemistry, Polymer Laboratory, University of Peshawar, Peshawar 25120, Pakistan; luqman_alisha@yahoo.com (L.A.S.); drrizwan@uop.edu.pk (R.U.); 2 Department of Chemistry, Shaheed Benazir Bhutto Women University, Peshawar 25000, Pakistan; 3 Department of Chemistry, University of Malakand, Chakdara 18800, Pakistan; sufaidkhan1984@uom.edu.pk; 4 Department of Chemistry, Quaid-i-Azam University, Islamabad 45320, Pakistan; m_sidiq12@yahoo.com; 5 Department of Chemistry, College of Science, Taif University, P.O. Box 11099, Taif 21944, Saudi Arabia; m.hessien@tu.edu.sa; 6 Department of Chemistry, Faculty of Science, Al-Azhar University, Nasr City, Cairo 11884, Egypt; zeinelbahy@azhar.edu.eg

**Keywords:** nanocomposites, multiwalled carbon nanotubes, poly(3-hydroxybutyrate), poly(methyl methacrylate), ammonia sensors, thermal stability, electrical properties

## Abstract

Partially biodegradable polymer nanocomposites Poly(3-Hydroxybutyrate) (PHB)/MultiwalledCarbon Nanotubes (MWCNTs)/Poly(Methyl Methacrylate) (PMMA)and non-biodegradable nanocomposites (MWCNTs/PMMA) were synthesized, and their thermal, electrical, and ammonia-sensing properties were compared. MWCNTs were chemically modified to ensure effective dispersion in the polymeric matrix. Pristine MWCNTs (p-MWCNTs) were functionalized with –COOH (a-MWCNTs) and amine groups (f-MWCNTs). Then, PHB grafted multiwalled carbon nanotubes (g-MWNTs) were prepared by a ‘grafting to’ technique. The p-MWCNTs, a-MWCNTs, f-MWCNTs, and g-MWCNTs were incorporated into the PMMA matrix and PMMA/PHB blend system by solution mixing. The PHB/f-MWCNTs/PMMA blend system showed good thermal properties among all synthesized nanocomposites. Results from TGA and dTGA analysis for PHB/f-MWCNTs/PMMA showed delay in T_5_ (about 127 °C), T_50_ (up to 126 °C), and T_max_ (up to 65 °C) as compared to neat PMMA. Higher values of frequency capacitance were observed in nanocomposites containing f-MWCNTs and g-MWCNTs as compared to nanocomposites containing p-MWCNTs and a-MWCNTs. This may be attributed to their excellent interaction and good dispersion in the polymeric blend. Analysis of ammonia gas-sensing data showed that PHB/g-MWCNTs/PMMA nanocomposites exhibited good sensitivity (≈100%) and excellent repeatability with a constant response. The calculated limit of detection (LOD) is 0.129 ppm for PHB/g-MWCNTs/PMMA, while that of all other nanocomposites is above 40 ppm.

## 1. Introduction

Polymers are versatile materials that have certain unique properties such as lowdensity, flexibility, toughness, easy processibility, and low conductivity [1]. However, these properties are still inadequate for efficient industrial applications. Hence, continuous research efforts are in progress to develop new polymeric materials with advanced properties. The desired properties can be obtained by adding filler (organic and inorganic materials) to the polymer matrix. Common fillers such as metal particles, silica, carbon black, etc. require high filler loading. High filler loading increases the costof composites along with challenging processibility. Nowadays, nanofillers are incorporated in polymer matrixes to introduce desired properties with low filler loading [1]. Nanofillers (size range: 1–100 nm) offer a range of excellent and adjustable physical and chemical properties [2]. One of the most extensively studied and used nanomaterials are carbon nanotubes (CNT). Extraordinary chemical and physical properties of the CNT (i.e. high stability, good conductivity, wide availability and easy functionalization) offer several advantages as compared to other commonly used materials [3,4]. CNTs are much sought after due to their nanoscale size, which confers exceptional thermal, electrical, mechanical, optical, and chemical capabilities [5].Conducting polymer nanocomposite is formed when the dispersed filler link through CNTs junctions within polymeric matrix. The creation of percolated pathways leads to the conduction of electrons and phonons. As a result, the composite transformed from an insulator to conductor [6]. Uniformly distributed CNTs in the polymeric matrix lowers the percolation threshold [7,8].

CNTs have been proved to be a potential gas sensing material for tiny, portable, and low-sensing devices in the preceding decade [9]. Their small size and high surface area provide a large number of sites for the adsorption of gas molecule, as well as their good electrical sensitivity, room temperature operation, and stability, making them a strong candidate for technological sensors [10]. Toxic gas sensing with semiconducting carbon nanotubes (as chemical and biological sensors) has been proposed [11]. Literature review shows that CNTs revealed good sensitivity to gases such as H_2_, NH_3_, NO_2_, SO_2_, CO, CH_4_, O_2_ and H_2_S. The adsorption of substrate molecules on the sidewalls of CNTs alters its electrical properties [12]. This change in electrical properties can be utilized as output signals in CNTs based sensors [13]. The major mechanism of sensing at the low temperature is electrical charge transfer among the gas molecules and MWCNTs [14]. Gases such as NH_3_, NO_2_, etc. adsorb directly on the surface of CNTs and cause the transfer of electrons, which changes the electrical conductivity of the nanotubes. To ameliorate the sensitivity characteristics of resistive gas sensors, the addition of polymers to carbon nanotubes is practiced widely these days. The van der Walls attraction between CNTs results in agglomerations [15,16]. Such agglomeration reduces the number of adsorption sites and the resulting gas sensors depict less sensitivity. The interfacial bonding between CNTs and the polymer is poor [17]. Agglomeration also disrupts percolation routes and degrades mechanical characteristics, resulting in a subpar composite [18,19,20]. The interaction of carbon nanotubes with the polymer matrix is required for synergistic effects [21]. This is favored by the chemical modification of MWCNTs with functional groups such as COOH, OH, and NH_3_. The newly introduced functional groups are capable of interaction with functional groups of the polymer matrix. These interactions can either be physical or chemical. Thus, the composites exhibit good interfacial adhesion and improved dispersion of modified MWCNTs in the polymeric matrix. Chemical functionalization can introduce desirable properties with respect to the amount and type of organic moieties deposited on the surface of CNTs [22]. Moreover, functionalized CNTs showed improved sensitivity and selectivity of the gas sensor [9].

In the last few years, biodegradable polymers obtained from renewable resources have gained significant consideration as a possible substitute for non-compostable polymers [23] which causes several environmental issues. In this study, poly(3-hydroxybutyrate) (PHB) has been selected, as a biodegradable polymer. PHB is water-insoluble and shows resistance to hydrolytic degradation [24]. These characteristics set PHB apart from the most currently used biodegradable polymers, which are either water-soluble or moisture sensitive. PHB is also biocompatible, non-toxic, and UV resistant, as well as having a high tensile strength (40 Mpa). These characteristics make PHB the best choice for a biodegradable sensor [25]. PHB is a delicate and rigid substance. When maintained at room temperature for an extended period of time, it becomes brittle. PHB’s brittleness and thermal instability make it difficult to use in a variety of applications [26]. The large crystal size of PHB carries poor mechanical properties [27]. Due to poor mechanical properties, limited functionalities, high manufacturing cost, thermal degradability just above the melting point, PHB needs modifications for enhanced performance in particular applications [28]. Frequent efforts were made to mix PHB with other flexible polymers or low atomic weight plasticizers to transform PHB into materials with enhanced mechanical strength and film formation [29,30]. Various researchers attempted to blend PHB with other polymers to attain the physical characteristics, and to reduce the fragility of PHB. Properties of PHB can be improved by blending it with Poly(methyl methacrylate) (PMMA). PMMA is a non-biodegradable thermoplastic. It is suitable for application in electrical engineering because of its good dielectric constant and low humidity/water absorption capability. Heat stabilized types can resist temperature up to 100 °C and show good resistance to temperature change [31]. PMMA has been selected due to its advantages such as low cost, easy molding and easy processing [32]. Abou [33] and Yong et. al. [34] indicated that a-PHB/PMMA blend shows improved miscibility when the content of PMMA is greater than 60 wt%.

Studies showed that the incorporation of functionalized MWCNTs even in a small amount can significantly improve the thermal, mechanical and electrical characteristics of PHB [35,36]. However, just a few studies on the preparation, characterization and properties of PHB based polymer nanocomposites have been reported. The bionanocomposites created by the uniform distribution of carbon-based nanofillers in a biopolymer matrix have a lot of potential [37,38,39]. The effect of MWCNTs functionalization on the thermal, electrical and ammonia-sensing capabilities of MWCNTs(4 wt%)/PMMA and PHB/MWCNTs(4 wt%)/PMMA thin films nanocomposites systems was investigated in this study.

## 2. Materials and Methods

### 2.1. Materials

MWCNTs (diameter: 80–100 nm), sulfuric acid (H_2_SO_4_ 98%), hydrochloric acid (HCl 37%), nitric acid (65%), chloroform (99.8%), ethanol (99.7%), tetrahydrofuran (THF), *N*.*N*-dimethylformamide (DMF) and 4,4′-diaminodiphenyl sulfone (DDS) (MW) ≈ 248.3 gmol^−1^) were purchased from Sigma Aldrich. PMMA (99.8% pure, average molecular weight (MW) ≈ 10,000 gmol^−1^) and PHB (98% pure, average molecular weight (MW) ≈ 760,000 gmol^−1^) were provided by BDH chemicals Ltd. Poole, England.

### 2.2. Functionalization of MWCNTs

p-MWCNTs and 3:1 solution of H_2_SO_4_ (8 M) and HNO3 (5 M) were refluxed at 60 °C for 5 h to introduce carboxylic group on the surface of MWCNTs. Acid functionalized MWCNTs (a-MWCNTs) thus obtained were filtered with a microfiltration membrane (0.4 μm). To attain neutral pH, the obtained a-MWCNTs were subsequently washed with distilled water [40]. The fabrication of amine-functionalized MWCNTs was performed by following a method developed by Freitas et al. [41] with slight modification. One g of the a-MWCNTs were treated with 10 g of DDS solution in *N*,*N*-dimethylformamide. The solution was refluxed at 65 °C for 98 h. The amine-functionalized MWCNTs (f-MWCNTs) that, formed were washed with THF after microfiltration to remove residual DDS and then were then dried for 7 h in a vacuum oven at 70 °C. For the synthesis of grafted or block polymeric structures with amphiphilic or biodegradable characteristics, a method developed by Yu et al [42] with slight modification was followed. The PHB and f-MWCNTs in a 1:2 ratio were refluxed in chloroform for 95 h at 62 °C. The g-MWCNTs obtained were filtered through a microfiltration membrane (0.4 μm) and were in a vacuum dried environment at 78 °C for 7 h.

### 2.3. Fabrication of Multiwalled Carbon Nanotubes (MWCNTs)/Polymethylmethacrylate (PMMA) Nanocomposite

The p-MWCNTs/PMMA, a-MWCNTs/PMMA, f-MWNTs/PMMA, andg-MWCNTs/PMMA nanocomposite were fabricated by solution casting method. First, 0.5 wt%, 1 wt%, 2 wt%, 4 wt%, 5 wt% and 10 wt% of each type of MWCNTs were added separately in 4 mL chloroform and sonicated for 2 h in separate conical flasks. In separate conical flasks, PMMA (2 g) in chloroform (6 mL) was stirred for 2 h. Then, the different weight fraction of each type of MWCNTs was added to PMMA and refluxed (at 60 °C for 96 h). After stirring, the samples were solution cast in clean and dry Petri dishes. The synthesized samples were vacuum dried in an oven until their weight was equilibrated and were then kept in a desiccator for minimal humidity effects.

### 2.4. Fabrication of Poly(3-Hydroxybutyrate) (PHB)/Multiwalled Carbon Nanotubes (MWCNTs)/Poly(Methyl Methacrylate) (PMMA)

The solubility limit of PHB with PMMA is 20% by weight. Excess PHB segregates and crystallizes [43], therefore, a different weight fraction of each type of MWCNTs was added in fixed composition of PMMA (80%) and PHB (20%) blend. A similar process was followed for the fabrication of PHB/p-MWCNTs/PMMA, PHB/a-MWCNTs/PMMA, PHB/f-MWCNTs/PMMA and PHB/g-MWCNTs/PMMA blend systems with 20% of PHB and 80% of PMMA as for MWCNTs/PMMA nanocomposites. However, it was observed that PHB/MWCNTs/PMMA samples containing only 4 wt% MWCNTs are capable of forming thin films. Therefore, all studies were performed on the PHB/MWCNTs/PMMA samples comprising 4 wt% of each type of MWCNTs.

### 2.5. Gas-Sensing Set-Up

The fabricated nanocomposites (0.05 μLdrop) were spin-coated (600 rpm) on interdigitated electrodes (line width: 210 μm, line space: 190 μm and size: 7 mm × 13.4 mm). The gas-sensing set-up was composed of an airtight jar (3.5 mL), a heating pad and a fan. When the system reached a steady state, it was exposed to different concentrations of ammonia at 25 °C with 60 ± 1% RH, Humidity was measured by using digital hygrometer (ExtechRH101, Shanghai, China). Ammonia evaporated with the help of a fan and heating pad. The resistance response was recorded using an LCR meter (HIOKI 3522-50 LCR HiTESTER, Nagano, Japan).

### 2.6. Material Characterization

FTIR spectra of pristine MWCNTs, functionalized MWCNTs, and thin-film nanocomposites were obtained by using anATR-FTIR (Shimadzu, Japan) within the region of 4000–500 cm^−1^ and with the resolution of 2 cm^−1^. For SEM images, a JSM-6490 (JEOL, Tokyo, Japan) electron microscope was used. Thermal degradation characteristics of all synthesized samples were performed using Parkin-Elmer, TGA-7 instrument (Parkin-Elmer, Waltham, MA, USA). TGA was performed at a scan rate of 10 °C/min over a temperature range of 25–600 °C. The AC conductance and capacitance values of all the synthesized nanocomposites were measured using Agilent LCR Meter E4980A (Agilent, Santa Clara, CA, USA) at room temperature (25 °C) with a voltage amplitude of 1.0 V. AC conductance and capacitance values were measured as a function of frequency. Measurements were made in the frequency range of 25 Hz to 2 MHz.

## 3. Results and Discussion

### 3.1. FTIR Analysis

**Figure 1:**
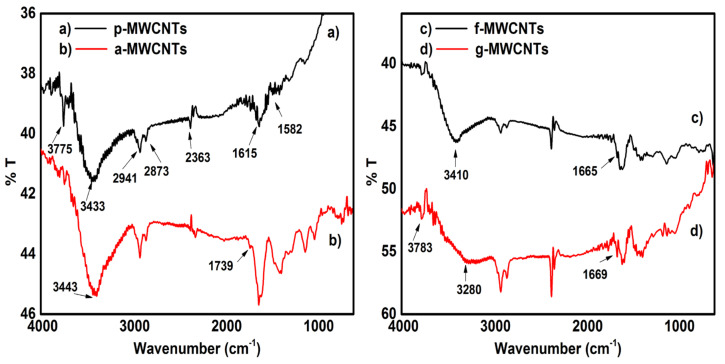
FTIR spectra of (**a**) p-MWCNTs, (**b**) a-MWCNTs, (**c**) f-MWCNTs, and (**d**) g-MWCNTs.

The FTIR spectra of pristine and functionalized MWCNTs are shown in Figure 1. The FTIR spectrum of pristine MWCNTs (Figure 1a) showed a skeletal vibration of aromatic rings (C=C stretching) at 1615 cm^−1^ and 1582 cm^−1^ which indicated the graphitic structure of MWCNTs [44]. In the spectrum of a-MWCNTs (Figure 1b), a new peak appeared at 1739 cm^−1^ in comparison to the spectrum of p-MWCNTs. This peak is attributed to the –C=O stretch of the -COOH group [45].

In the FTIR spectrum of f-MWCNTs, (Figure 1c) a hump appeared at 1665 cm^−1^, that corresponds to the C=O stretch of the amide structure [40]. A band at 3410 cm^−1^ corresponds to the stretching vibrations of –N-H and –OH [46]. Peaks at 1455 cm^−1^ and 1277 cm^−1^ were attributed to the stretching vibration of C–N in the amide groups. The peaks at 1636 cm^−1^ and 1357 cm^−1^ were assigned to the N-H bending and to the C-N stretching of amine, respectively [47]. A peak at 1636 cm^−1^ shows N-H deformation in primary amines [48]. A peak at 623 cm^−1^ corresponds to the amide structure N-C=O. In the FTIR spectrum of g-MWCNTs (Figure 1d), a peak appeared at 1162 cm^−1^, which corresponds to the C-O-C stretch of grafted PHB. An increase in the peaks intensity at 2920 and 2857 cm^−1^ is due to a large number of the C-H bonds of grafted PHB chains. An increase in the peak intensity at 1669 cm^−1^ (C(=O)NH stretching) and 1552 cm^−1^ (N-H bending) in g-MWCNTs as com-pared to f-MWCNTs shows an increase in the already existing amide bonds due to grafting of PHB polymer on f-MWCNTs [49]. The FTIR spectra and interpretation of MWCNTs(4 wt%)/PMMA and PHB/MWCNTs(4 wt%)/PMMA based nanocomposites have been described in the Supplementary Materials, Text S1 and Figures S1–S3.

### 3.2. Morphological Characterization

**Figure 2:**
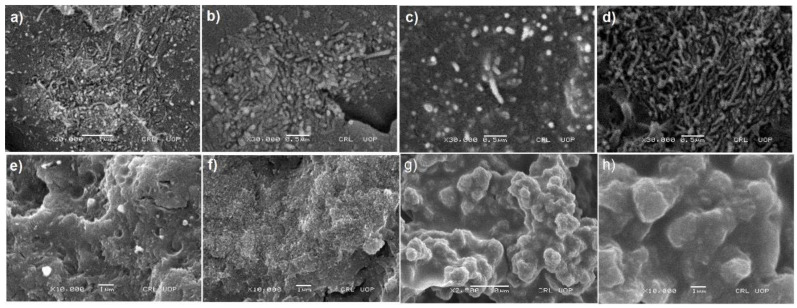
SEM images of (**a**) p-MWCNTs(4 wt%)/PMMA, (**b**) a-MWCNTs(4 wt%)/PMMA, (**c**) f-MWCNTs(4 wt%)/PMMA, (**d**) g-MWCNTs(4 wt%)/PMMA (**e**) PMMA/p-MWCNTs(4 wt%)/PHB, (**f**) PMMA/a-MWCNTs(4 wt%)/PHB, (**g**) PMMA/f-MWCNTs(4 wt%)/PHB and, (**h**) PMMA/g-MWCNTs(4 wt%)/PHB.

SEM image of p-MWCNTs shows the characteristic typical aggregates for MWCNTs structures due to Van der Waals interactions among the tubes (see Supplementary Materials, Figure S4a). However, g-MWCNTs shows entangled thread like structure, which is the characteristic of functionalization (see Supplementary Materials, Figure S4b). Functionalization reduced the aggregates formation. SEM images of fractured surface thin filmed nanocomposites containing p-MWCNTs, a-MWCNTs, f-MWCNTs and g-MWCNTs in the PMMA matrix is shown in Figure 2a–d respectively. Figure 2a showed that p-MWCNTs agglomerated in the PMMA matrix owing to strong van der Waals forces. The smooth-surface of p-MWCNTs exhibits weak interaction with polymer [50]. The nanocomposites comprising of a-MWCNTs and g-MWCNTs [Figure 2b,d] showed no substantial enhancementin the morphology. However, in the nanocomposites containing f-MWCNTs, the dispersion has been considerably homogenized. The fractured surfaced SEM image in Figure 2c shows uniformly dispersed tips of untangled f-MWCNTs in PMMA matrix. Amine groups on f-MWCNTs exhibit strong interfacial interaction with PMMA. SEM images of the cryofractured surface of nanocomposites containing p-MWCNTs, a-MWCNTs, f-MWCNTs and g-MWCNTs in PHB/PMMA blend are presented in Figure 2e–h, respectively. The surface morphology changes on the addition of PHB to PMMA/MWCNTs nanocomposites. Figure 2f–e shows the development of surface morphology from smooth to rough on the addition of PHB to p-MWCNTs(4 wt%)/PMMA and a-MWCNTs(4 wt%)/PMMA nanocomposites. By the addition of f-MWCNTs (4 wt%) [Figure 2g] and g-MWCNTs (4 wt%) [Figure 2h] in the blend system of PHB/PMMA sponge-like pores appear in the membrane. In Figure 2g,h, the brightest areas show the highest point in the cross-section of the solution cast film and the dark areas indicate the valleys or pores. Thus, it can be inferred that in nanocomposites of PHB/f-MWCNTs(4 wt%)/PMMA and PHB/g-MWCNTs(4 wt%)/PMMA, the PHB/PMMA may wrap around f-MWCNTs and g-MWCNTs, respectively to prevent their aggregation and it is hard to see these nanotubes with in the micrographs.

### 3.3. Electrical Properties

**Figure 3:**
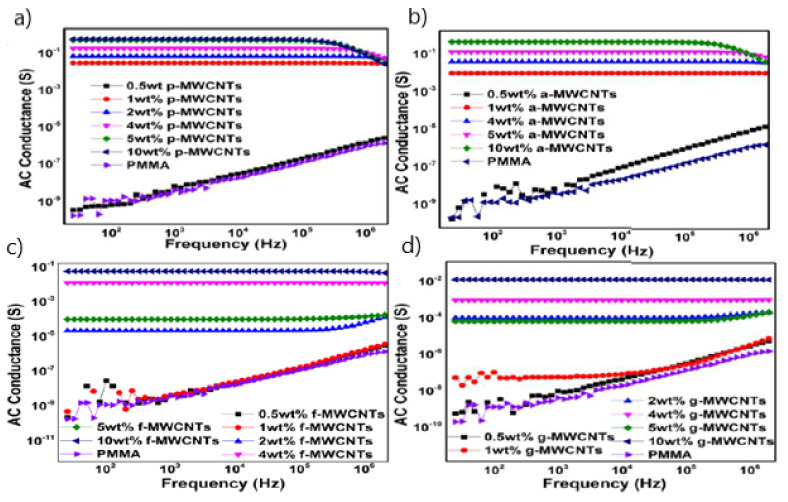
AC electrical conductance versus frequency for (**a**) p-MWCNTs/PMMA (**b**) a-MWCNTs/PMMA (**c**) f-MWCNTs/PMMA and (**d**) g-MWCNTs/PMMA nanocomposites as a function of different weight fractions of p-MWCNTs, a-MWCNTs, f-MWCNTs and g-MWCNTs, respectively.

AC electrical conductance for p-MWCNTs/PMMA, a-MWCNTs/PMMA, f-MWCNTs/PMMA, g-MWCNTs/PMMA, and neat PMMA as a function of frequency for different weight fractions of p-MWCNTs, a-MWCNTs, f-MWCNTs and g-MWCNTs is presented in Figure 3A–D, respectively. The AC conductivity of the nanocomposites provides information of the connectivity and electron transport mechanism of the MWCNTs network in the polymeric matrix (see Supplementary Materials, Text S2) [51,52,53].

The composite containing 0.5 wt% p-MWCNTs in PMMA matrix (Figure 3A) exhibits frequency-dependent conductance and thus remains insulated and lies below the percolation threshold. The behaviour of the 0.5 wt% nanocomposite is essentially similar to the PMMA matrix, with the exception that its conductance is an order of magnitude larger. At low filler loading, the filler content is not sufficient to form a conductive network, therefore, AC conduction occurred through the tunneling current. Figure 3A shows that in nanocomposite containing 1% p-MWCNTs, there is a significant increase in conductance from 1.733 × 10^−10^ to 2.817 × 10^−2^ S at a frequency of 25 Hz. The increase in the AC conductivity is about 8 order of magnitude which indicates that the percolation threshold is between p-MWCNTs weight fraction of 0.5 and 1 wt%. When the p-MWCNTs weight fraction is above the percolation threshold, the nanotubes interconnected and formed the conductive pathways, and the leakage current is responsible for the increase in the AC conductance [54]. Thus, the addition of 1% p-MWCNTs into the PMMA matrix is sufficient to obtain nanocomposites requiring electrical discharge. It can be observed that AC conductance becomes frequency independent after percolation has been attained. The high aspect ratio of MWCNTs allowed the achievement of the percolation threshold at a low weight fraction. When the percolation threshold is reached, the rise in conductance is relatively small, and an electrical conductivity plateau is obtained on further increasing the wt% of p-MWCNTs in subsequent nanocomposites. As the p-MWCNTs concentration in nanocomposites increases from 0 to 10 wt%, the conductance rises by nine orders of magnitude. The increasing weight fraction of nanotubes reduced the intertube distance, thereby allowed effective charge transport [55]. However, in the high-frequency region, the conductance of the samples (i.e., 5 and 10 wt% p-MWCNTs/PMMA) was found to decrease with an increase in the frequency. In this high-frequency region, the electron hopping processmay be obstructed by the applied field [56,57], or the drop in the high-frequency region may also indicate that the measurements were outside the upper experimental range of the LCR meter used.

The result for AC electrical conductance versus frequency for a-MWCNTs/PMMA nanocomposites of different weight percentages of a-MWCNTs is presented in Figure 3B. The same frequency dependence behavior is seen for a sample containing below 1 wt% a-MWCNTs. At 0.5 wt%, the nanocomposite remains an insulator. A significant increase in conductance was observed at 1 wt% p-MWCNTs/PMMA sample. The increase in the electrical conductance was 7 orders of magnitude, from 1.733 × 10^−10^ to 8 × 10^−3^ S for 1 wt% a-MWCNTs/PMMA sample relative to neat PMMA at 25 Hz frequency, which corresponds to DC electrical conductance. When the weight fraction increased from 1 to 5 wt%, an increase of 2 orders was found. As the concentration of a-MWCNTs increases from 0 to 10 wt% in the nanocomposite, the total increase in conductance was 9 orders of magnitude.

The AC electrical conductance vs. frequency of f-MWCNTs/PMMA nanocomposites for various weight fractions of f-MWCNTs is demonstrated in Figure 3C. The figure showed that nanocomposites with 0.5 and 1 wt% f-MWCNTs lie below the percolation threshold and remain nonconductive as they exhibit frequency-dependent behavior. The nanocomposite with 2 wt% f-MWCNTs exhibits a significant increase in electrical conductance from 1.733× 10^−10^ to 2.0× 10^−5^ S at a frequency of 25 Hz, which is about five orders of magnitude. The total increase in electrical conductance (as the weight fractions of f-MWCNTs increases from 0 to 10 wt%) was eight orders of magnitude. The conductance values of the nanocomposite containing 2 wt% f-MWCNTs do not vary (stay constant) with increasing frequency up to a specified frequency in the high-frequency region beyond which an increase in electrical conductance was observed. Such behavior can be explained by modeling the complex admittance, Y, as a capacitor and resistor in parallel as defined by Equation (1).

(1) $$Y\left(\omega \right)=Y^{\prime }+Y^{\prime \prime }=\frac{1}{R}+j\omega C\;$$

where *R* is the resistance, *C* is the capacitance, and *ω* is the angular frequency (*ω* = 2π*f*), where *f* is frequency. The AC electrical conductivity *σ*_AC_(*ω*) is equal to Equation (2).

(2) $$\sigma_{\mathrm{AC}}\left(\omega \right)=\left|Y\left(\omega \right)\right|\frac{t}{A}$$

where *t* is the thickness of the sample and *A* is the area of the electrode. The DC electrical conductivity, *σ*_DC_, is given by Equation (3).

(3) $$\sigma_{\mathrm{DC}}=Y^{\prime \frac{t}{A}}$$

The result in Figure 3C can be explained by the resistor-capacitor model. The above equations show that the ohmic behavior, 1/*R*, and the frequency induced dielectric behavior, *jωC*, contribute to the total AC electrical conductance. When the weight fraction of f-MWCNTs is below the percolation threshold, the impact of *Y*′ is very reduced due to a lack of conductivity networks, and hence *Y*″ controls the AC conductivity. The dielectric material acts as a capacitor due to this frequency dependency. After percolation, the charge carriers begin to flow across the conductive networks and ohmic behavior takes over [53,58]. As a result, the real component of admittance controls the conductivity, while the influence of frequency is only significant at higher frequency levels.

**Figure 4:**
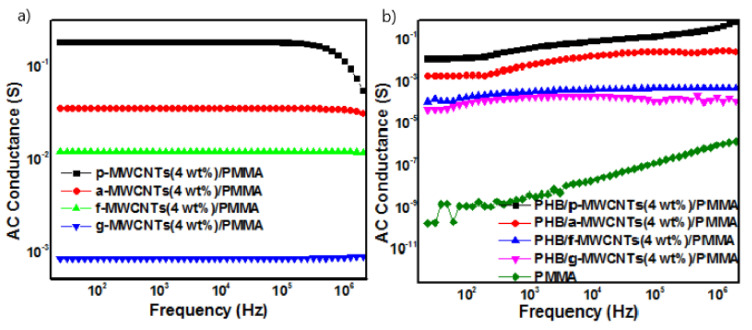
AC electrical conductance as a function of frequency for (**a**) MWCNTs(4 wt%)/PMMA nanocomposites. (**b**) variation of AC conductance with frequency for PHB/MWCNTs(4 wt%)/PMMA nanocomposites.

The variation in AC conductance of g-MWCNTs/PMMA nanocomposites with frequency for various weight fractions of g-MWCNTs is presented in Figure 3D. The nanocomposites containing 0.5 and 1 wt% g-MWCNTs remain nonconducting and exhibit frequency-dependent behavior. A significant increase of five orders of magnitude was found in electrical conductance for a sample containing 2 wt% g-MWCNTs as compared to neat PMMA, i.e., from 1.733 × 10^−10^ to 8.8× 10^−5^ S at 25 Hz. This indicates that the percolation threshold is between 1 and 2 wt% samples. As the concentration increases from 0 to 10% g-MWCNTs, the total increase in conductance was about eight orders of magnitude. Figure 4A shows the comparative behavior of the variation of electrical conductance with frequency for p-MWCNTs(4 wt%)/PMMA, a-MWCNTs(4wt%)/PMMA, f-MWCNTs(4 wt%)/PMMA, and g-MWCNTs(4 wt%)/PMMA nanocomposites. The g-MWCNTs exhibited the lowest values of electrical conductivity, which may be attributed to the coating of the individual nanotubes by a grafted polymer. MWCNTs coated with the insulating surface not only contribute to dispersion but also result in poor electrical contact between nanotubes.

The comparative variation of AC conductance with frequency for PHB/p-MWCNTs(4 wt%)/PMMA, PHB/a-MWCNTs(4 wt%)/PMMA, PHB/f-MWCNTs(4 wt%)/PMMA and PHB/g-MWCNTs(4 wt%)/PMMA nanocomposites are shown in Figure 4B. The addition of PHB to MWCNTs results in an insulating covering. As a result, the barrier gap for MWCNTs widens even more. Consequently, MWCNTs cannot connect directly to each other, which induces the lower AC conductance of PHB/MWCNTs(4 wt%)/PMMA than MWCNTs(4 wt%)/PMMA nanocomposites. The composites incorporating f-MWCNTs and g-MWCNTs had the lowest AC conductance values of all the nanocomposites examined (see Supplementary Materials, Table S1). This is likely due to their good interaction and dispersion in the polymeric blend. These findings are also supported by FTIR and SEM research.

#### Energy Storage Properties 

**Figure 5:**
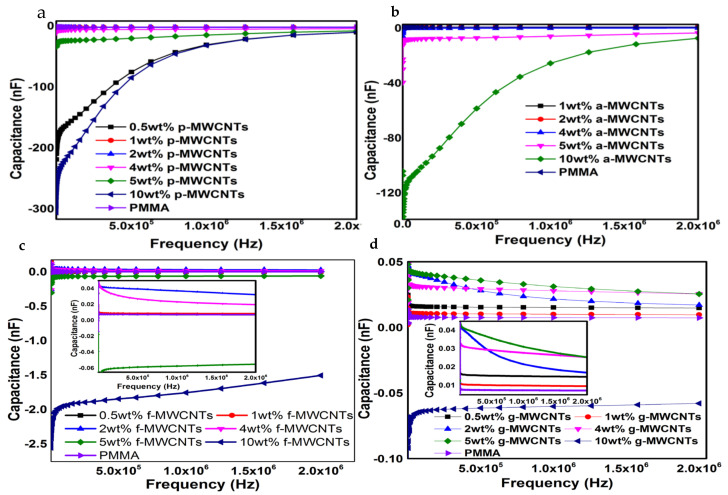
Capacitance versus frequency for (**a**) p-MWCNTs/PMMA (**b**) a-MWCNTs/PMMA (**c**) f-MWCNTs/PMMA and (**d**) g-MWCNTs/PMMA at different weight fractions of p-MWCNTs, a-MWCNTs, f-MWCNTs, and g-MWCNTs/PMMA.

**Figure 6:**
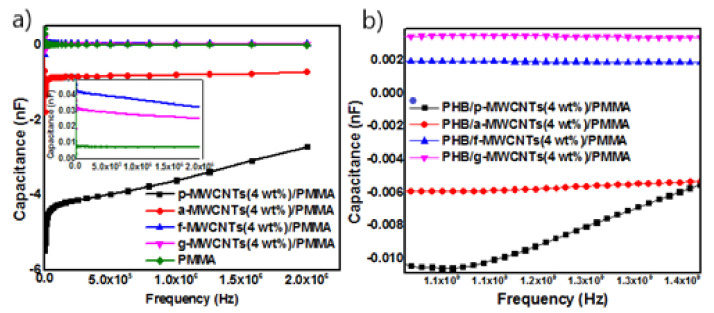
Capacitance as a function of frequency for (**a**) MWCNTs(4 wt%)/PMMA and (**b**) 411 PHB/MWCNTs(4 wt%)/PMMA.

The ability of the synthesized nanocomposites to store energy in the form of electric charge can be measured with respect to their capacitance. All MWCNTs/PMMA containing p-MWCNTs and a-MWCNTs exhibit lower capacitance than the capacitance of neat PMMA [Figure 5a,b]. However, in f-MWCNTs/PMMA, the capacitance values of all composites containing f-MWCNTs are higher than neat PMMA except for 5 wt% and 10 wt% f-MWCNTs/PMMA nanocomposites (Figure 5c). In g-MWCNTs/PMMA nanocomposites the capacitance values of all studied compositions are higher than the neat PMMA except for the nanocomposite containing 10 wt% g-MWCNTs (Figure 5d). The variation of capacitance with frequency for p-MWCNTs(4 wt%)/PMMA, a-MWCNTs(4 wt%)/PMMA, f-MWCNTs(4 wt%)/PMMA and g-MWCNTs(4 wt%)/PMMA depicts that among all nanocomposites containing differently functionalized MWCNTs, f-MWCNTs(4 wt%)/PMMA exhibits higher capacitance values than that of PMMA (Figure 6a). The frequency capacitance of the PHB/p-MWCNTs(4 wt%)/PMMA, PHB/a-MWCNTs(4 wt%)/PMMA, PHB/f-MWCNTs(4 wt%)/PMMA and PHB/g-MWCNTs(4 wt%)/PMMA are compared in Figure 6b. The capacitance values of PHB/MWCNTs/PMMA show that the composites containing f-MWCNTs and g-MWCNTs exhibit slightly higher capacitance values. The composites with capacitance values higher than neat PMMA are capable of storing electrical energy and can be used in electronic power devices [59]. The capacitance values of 2 wt% and 5 wt% g-MWCNTs/PMMA and 5 wt% f-MWCNTs/PMMA nanocomposites showed a decreasing trend with increasing frequency. The decrease in capacitance is because dipoles’ relaxation is unable to reach equilibrium with electric field variation at different frequencies [60]. However, the increase in the capacitance with frequency may be due to the development of microcapacitors in the polymer matrix of dielectric materials when neighboring nanotubes are disconnected by a thin polymer [61]. Figure 5d depicted that increasing the wt% of g-MWCNTs in the PMMA matrix increases the microcapacitors formation with an increase in effective capacitance. This enhancement may be due to the polarization effects through the buildup of charges at the interfaces of the heterogeneous dielectric material according to Maxwell Wagner Sillar’s theory. However, the lower capacitance value of g-MWCNTs(10 wt%)/PMMA than neat PMMA may be due to the agglomeration of MWCNTs in the polymeric matrix. The creation of the conductive network grows as the filler content increases, resulting in substantial current leakage. Nanocomposites including f-MWCNTs (Figure 5c) and g-MWCNTs (Figure 5d) have higher frequency capacitance than nanocomposites containing p-MWCNTs (Figure 5a) and a-MWCNTs (Figure 5b). This emphasizes the importance of uniform dispersion and strong interfacial adhesion, which may be achieved by modifying MWCNTs, for nanocomposites to have significant energy storage capacity. However, the addition of PHB to MWCNTs/PMMA nanocomposites (Figure 6b) had no discernible influence on capacitance values.

### 3.4. Thermogravimetric Analysis (TGA)

**Figure 7:**
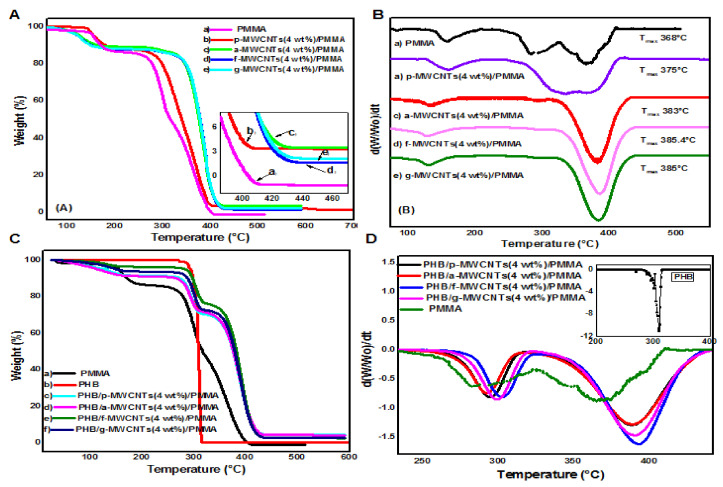
Comparison of (**A**) TGA and (**B**) dTGA curves for PMMA and PMMA(4 wt%)/MWCNTs nanocomposites. (**C**) TGA and (**D**) dTGA profiles of PMMA and PHB/MWCNTs(4 wt%)/PMMA nanocomposites. The inset graph shows the dTGA curve of PHB.

The TGA and dTGA curves for PMMA and MWCNTs(4 wt%)/PMMA nanocomposites are presented in Figure 7A,B. The derivative thermogravimetric (dTGA) results were acquired by taking the time derivative of the ratio of the sample weight (W) to the initial sample weight (Wₒ), d(W/Wₒ)/dt [62]. Figure 7B shows that the complete degradation of PMMA occurs at ≈ 396 °C. In dTGA of PMMA two minor degradation steps appeared at 157 and 287 °C. A major degradation step also appeared at 368 °C. This result is consistent with earlier published data [63,64,65]. The first peak corresponds to the decomposition of the weak head to head bonds arising from chain termination by the combination [66]. It may also predict the presence of solvent in polymer film [67]. The second one may correspond to the transfer of radicals tomonomers [68]. The third peak of thermal decomposition is due to the random scission of the PMMA backbone [62,69]. The peak height increases with an increase in temperature [65].

Different parameters regarding thermal stability were calculated such as temperature at 5% weight loss (T_5_), temperature at 10% weight loss (T_10_), temperature at 50% weight loss (T_50_), maximum degradation temperature (T_max_), complete degradation temperature (T_end_) and residual material at 400 °C. The relative thermal stability of all synthesized nanocomposites was assessed by comparing T_5_, T_10_, T_50_, T_max_, T_end_ and the amount of residual material remaining at 400 °C. Thermogravimetric data analyses of all the synthesized nanocomposites along with neat PMMA and PHB are reported in Table 1. Data analysis showed that the addition of pristine MWCNTs, as well as differently functionalized MWCNTs in PMMA alone and in the PHB/PMMA blend system, increases its thermal stability. T_50_, T_max_ and T_end_ for nearly all the composites is shifted towards high temperature as compared to neat PMMA and PHB. Polymer chains may degrade more slowly near MWCNTs, which may delay the degradation temperature to high values. The thermal stability of nanocomposites is enhanced due to the greater thermal conductivity of MWCNTs, which promotes heat dissipation in the nanocomposites [70].

The dTGA curves (Figure 7B(b–e)) of MWCNTs(4 wt%)/PMMA nanocomposites show strong suppression of the peak at 287 °C. This indicates that in the presence of MWCNTs, the transfer of PMMA radicals to monomers is significantly suppressed. The data in Table 1 shows that the addition of f-MWCNTs and g-MWCNTs to the PMMA matrix has a substantial effect on the improvement of thermal stability at T_50_ (up to 61 degrees). An increase in the temperature at complete degradation (about 18 degrees) is shown in an expanded view of the TGA curves (380–460 °C) in Figure 7A(f,g). The good thermal stability of modified MWCNTs based nanocomposites is associated with their better dispersion in the matrix PMMA since it hinders the flux of degradation product and hence delays the decomposition process. It may be concluded that the thermal stability depends upon the difference in the extent of interaction between PMMA and modified MWCNTs, consequently. After complete decomposition, all the organiccontent removes from the sample and only carbon nanotube with a small amount of residue is left. The data given in Table 1 shows that the degradation temperature of f-MWCNTs(4 wt%)/PMMA and g-MWCNTs(4 wt%)/PMMA based nanocomposite at 5% weight loss (T_5_) and 10% weight loss (T_10_) shifted towards lower temperature values (up to 31 degrees). This reduction in T_5_ and T_10_ values are supported by the structural defects on the surface of MWCNTs upon functionalization as well as thermal degradation of carboxylic acid groups and 4,4-diaminodiphenyl sulphone moieties besides the decomposition of weak linkages in PMMA.

Figure 7C,D show TGA and dTGA curves for PMMA, PHB and PHB/MWCNTs(4 wt%)/PMMA nanocomposites. Figure 7C(b) shows that PHB decomposes in a single stage between 274 and 315 °C, with strong molecular structural stability up to 260 °C. The second step in TGA curves (Figure 7C(c–f)) for all PHB/MWCNTs(4 wt%)/PMMA nanocomposites indicates the thermal degradation of PHB. Figure 7D(a–d) shows the evident shift in dTGA curves of PHB/MWCNTs(4 wt%)/PMMA composites towards high temperature as compared to neat PMMA (Figure 7D(e)). Data in Table 1 depict that incorporation of PHB further improves the thermal stability of nanocomposites. However, PHB/f-MWCNTs(4 wt%)/PMMA blend system shows good thermal properties among all the synthesized nanocomposites. In comparison to neat PMMA, results from TGA and dTGA analyses of PHB/f-MWCNTs(4 wt%)/PMMA reveal a delay in T_5_ (about 127 °C), T_50_ (up to 126 °C) and T_max_ (up to 65 °C) in contrast to. The improved thermal properties may correspond to good miscibility and compatibility of f-MWCNTs in both polymers (i.e. PMMA and PHB). The compatibilization occurred by forming covalent bonds. Secondary interactions such as hydrogen bonding between the components of blend also played their role in imparting compatibilization. Secondary interaction may occur because of interactive functional groups introduced on the sidewalls of MWCNTs [71]. The hydrophobic nature of PHB could explain the highest delay in T_5_ and T_10_. In its molecular structure, PHB absorbs the least quantity of moisture [72]. This hydrophobic property is especially noticeable in composites with high MWCNTs dispersion. As a result, MWCNTs are surrounded by polymer chains, reducing the weight loss caused by moisture. However, the little proportion of weight may be related to the deterioration of PMMA’s poor head-to-head connections.

### 3.5. Analysis of Ammonia Gas Sensing Properties

The sensitivity (S) can be determined by following Equation (4).

(4) $$S=\frac{Ri-R{}^{\circ}}{R{}^{\circ}}\times 100$$

where *Ri* is the resistance of the sensor in ammonia and *R*° is the resistance of the sensor in the air. The response and recovery time is defined as the time of 90% total sensor’s resistance change.

LOD is calculated by using Equation (5).

LOD = 3S_D_/m (5)

where m is the slope of calibration curve. S_D_ corresponds to the standard deviation of noise in the response curve in dry air.

#### 3.5.1. PMMA/MWCNTs Nanocomposites based Ammonia Gas Sensors

**Figure 8:**
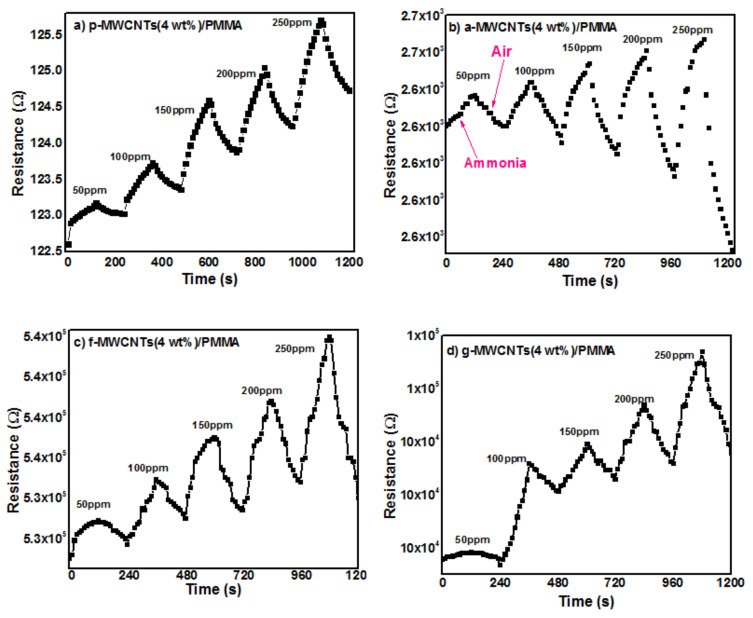
Response of (**a**) p-MWCNTs(4 wt%)/PMMA, (**b**) a-MWCNTs(4 wt%)/PMMA, (**c**) f-MWCNTs(4 wt%)/PMMA and (**d**) g-MWCNTs(4 wt%)/PMMA nanocomposites to ammonia va-pors.

**Figure 9:**
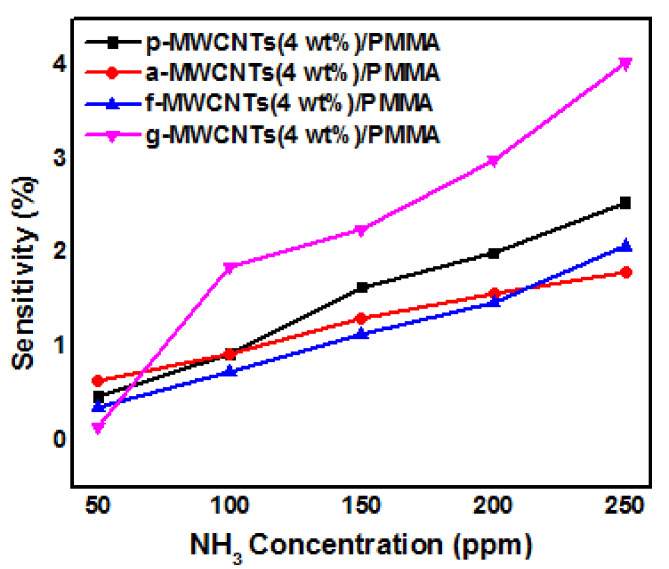
Sensitivity of p-MWCNTs(4 wt%)/PMMA (black line), a-MWCNTs(4 wt%)/PMMA (red line), f-MWCNTs(4 wt%)/PMMA (blue line) and g-MWCNTs(4 wt%)/PMMA (pink line) as a function of NH_3_ concentration.

**Figure 10:**
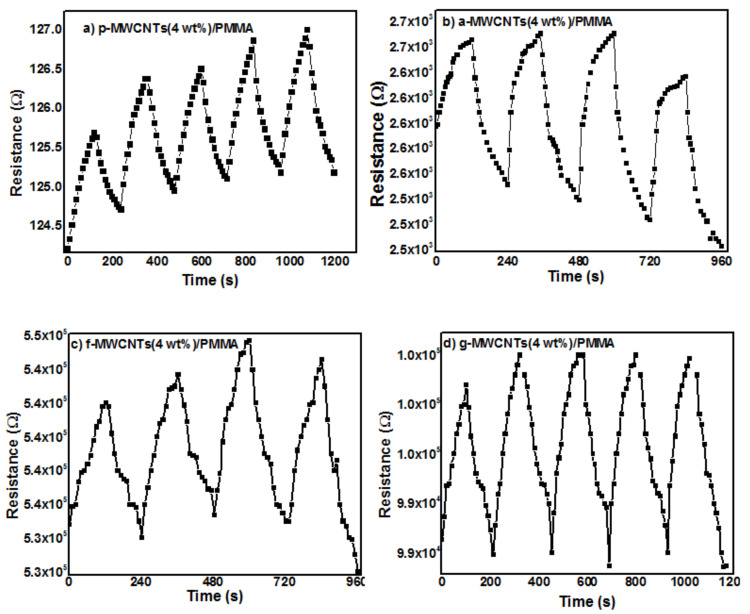
Cyclability of (**a**) p -MWCNTs(4 wt%)/PMMA, (**b**) a-MWCNTs(4 wt%)/PMMA, (**c**) f-MWCNTs(4 wt%)/PMMA and (**d**) g-MWCNTs(4 wt%)/PMMA nanocomposites to ammonia vapors at 250 ppm.

The p-MWCNTs(4 wt%)/PMMA, a-MWCNTs(4 wt%)/PMMA, f-MWCNTs(4 wt%)/PMMA and g-MWCNTs(4 wt%)/PMMA sensors were exposed to increasing concentrations of ammonia vapors. An increase in the electrical resistance was observed as shown in Figure 8a–d. This increase in the electrical resistance is attributed to the fact that polar NH_3_ molecules interact through hydrogen bonding with oxygen functionalities present on the sidewall MWCNTs. Ammonia is a reducing gas and transfers electron(s) to the p-type MWCNTs. The transferred electron(s) recombines with ‘hole’ carriers in p-type MWCNTs, depleting charge carrier holes and raising the electrical resistance [73]. Due to the adsorption of gas molecules, the volume of the PMMA matrix changes which inturn increases the intertube distance. As a result, the resistance increases [74]. The higher sensitivity of the g-MWCNTs(4 wt%)/PMMA sensor can be attributed to the greater interaction of ammonia with g-MWCNTs, as shown in Figure 9. Figure 10a–d show the response of the gas sensors after being exposed to ammonia gas periodically. The g-MWCNTs(4 wt%)/PMMA nanocomposites [Figure 10d] had a good reversible reaction to ammonia vapors.

#### 3.5.2. PMMA/MWCNTs(4 wt%)/PHB Nanocomposites based Ammonia Gas Sensors

**Figure 11:**
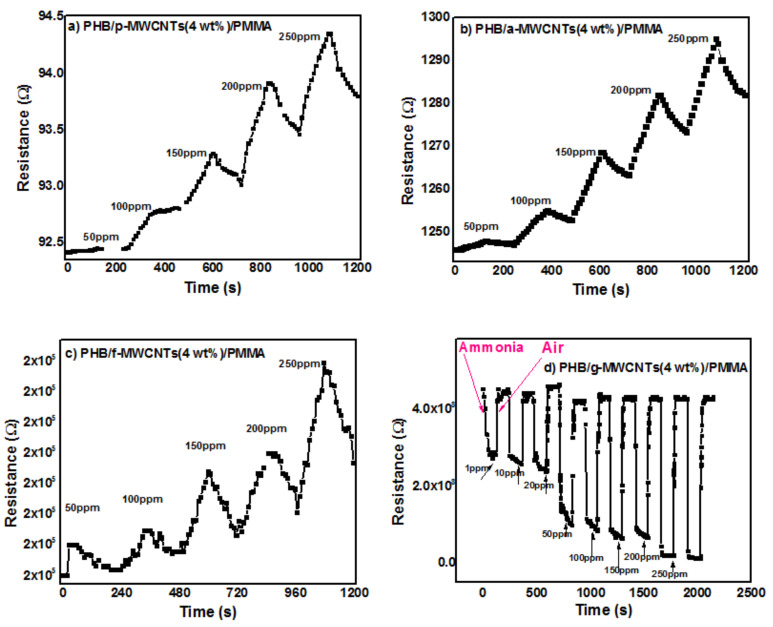
Response of (**a**) PHB/p-MWCNTs(4 wt%)/PMMA, (**b**) PHB/a-MWCNTs(4 wt%)/PMMA, (**c**) PHB/f-MWCNTs(4 wt%)/PMMA and (**d**) PHB/g-MWCNTs(4 wt%)/PMMA nanocomposites to ammonia vapors.

The response of PHB/p-MWCNTs(4 wt%)/PMMA, PHB/a-MWCNTs(4 wt%)/PMMA, PHB/f-MWCNTs(4 wt%)/PMMA and PHB/g-MWCNTs(4 wt%)/PMMA sensors to various concentrations of ammonia are shown in Figure 11a–d. Except for PHB/g-MWCNTs(4 wt%)/PMMA sensor, all the synthesized nanocomposites showed an increase in resistance following exposure to ammonia (Figure 11a–c). When exposed to ammonia, the PHB/g-MWCNTs(4 wt%)/PMMA (Figure 11d) sensor displayed a decreasing trend in resistance, which is trivial for an n-type semiconductor. Both the amine groups [75] and PHB [76] act as electron donors, effectively converting MWCNTs to n-type semiconductors. Since g-MWCNTs are uniformly dispersed in the polymeric matrix, they interact well with PHB, resulting in a greater number of g-MWCNTs coming into physical contact with the electron-donating PHB molecules, allowing for more effective doping. In n-type semiconductors, electrons are the primary charge carriers [77]. The sensor material adsorbed oxygen molecules from the air (Equation (6)). The charge carriers (i.e., electrons) in n-type semiconductors supports the formation of charged oxygen species.

(6) $$O_{2(\mathrm{gas})}+2e\to 2{O^{-}}_{\left(\mathrm{adsorbed}\right)}$$

(7) $${\mathrm{NH}}_{3(\mathrm{adsorbed})}+{O^{-}}_{\left(\mathrm{adsorbed}\right)}\to {[{\mathrm{NH}}_{2.}H-O{]}^{-}}_{\left(\mathrm{adsorbed}\right)}$$

(8) $$2{[{\mathrm{NH}}_{2.}H-O{]}^{-}}_{\left(\mathrm{adsorbed}\right)}+{O^{-}}_{\left(\mathrm{adsorbed}\right)}\to N_{2}+3H_{2}O+3e^{-.}$$

However, depletion of electron concentration increases resistance. Ammonia onexposure to PHB/g-MWCNTs(4 wt%)/PMMA, interacts with its lone pair of electrons to the interacting sites present in the porous surface [78]. Then, the absorbed ammonia molecules interact with charged oxygen species subsequently, releasing N2 and water (Equations (7,8)). The captured electrons are released into the conduction band, resulting in an increase in the concentration of charge carriers in the sample. The increase in charge carrier concentration is proportional to the decrease in the resistance [79].

**Figure 12:**
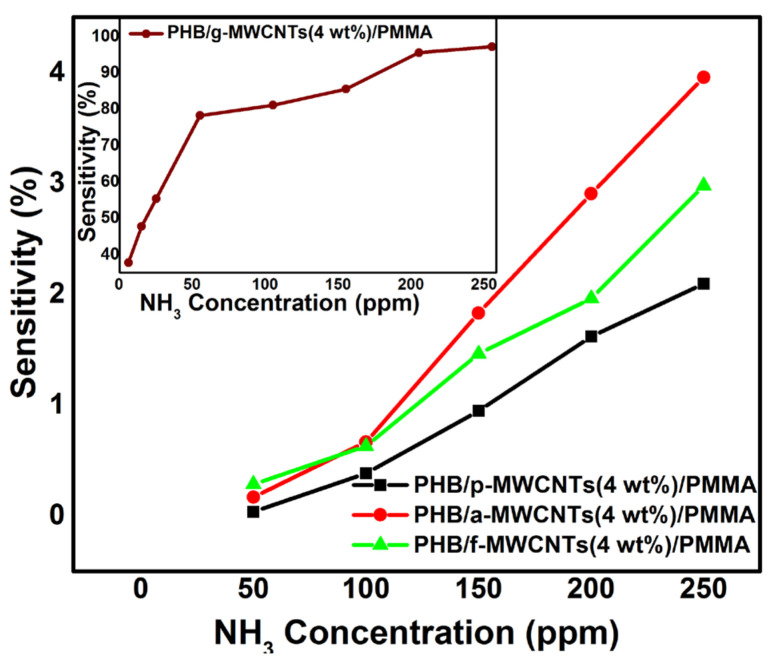
Sensitivity of PHB/p-MWCNTs(4 wt%)/PMMA (black line), PHB/a-MWCNTs(4 wt%)/PMMA (red line) and PHB/f-MWCNTs(4 wt%)/PMMA (green line). Inset shows the sensitivity of PHB/g-MWCNTs(4 wt%)/PMMA (brown line) as a function of NH_3_ concentration.

**Figure 13:**
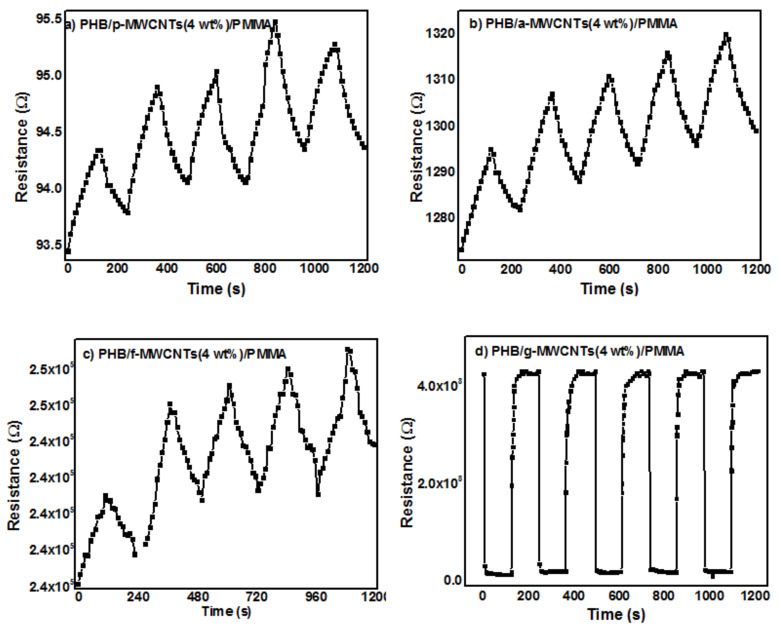
Cyclability of (**a**) PHB/p-MWCNTs(4 wt%)/PMMA, (**b**) PHB/a-MWCNTs(4 wt%)/PMMA, (**c**) PHB/f-MWCNTs(4 wt%)/PMMA and (**d**) PHB/g-MWCNTs(4 wt%)/PMMA nanocomposites to ammonia vapors at 250 ppm.

SEM studies revealed the highly porous morphology of PHB/g-MWCNTs(4 wt%)/PMMA nanocomposites. Due to the porous nature of the sample, gas can diffuse into and out of the nanocomposites quickly and shortens the response and recovery time consequently. The enhanced performance of PHB/g-MWCNTs(4 wt%)/PMMA in the response time and sensitivity was due to its higher effective surface area. The gas carriers also desorbed the analyte from the porous structure more quickly, as shown in Figure 11d. LOD calculated for PHB/g-MWCNTs(4 wt%)/PMMA is 0.129 ppm while the detection limit of all other nanocomposites is above 40 ppm. PHB/g-MWCNTs(4 wt%)/PMMA showed linear sinsitivity in 1–50 ppm range. The performance of the PHB/g-MWCNTs(4 wt%)/PMMA sensor outperforms that of earlier MWCNTs-based composites. Sahal et al. [80] reported a linear sensitivity of 5–20 ppm for a PANI/PMMA/PS/MWCNTs ammonia sensor with 0.830 ppm LOD. Marity and Ramasamy et al. [81] synthesized MWCNTs/PANI fabric sensor and reported 0.200 ppm LOD. For HCSA-doped PANI:CNTs, Marcelo and Carlos et al. [82] reported 4 ppm LOD. The sensitivity comparision of the PHB/p-MWCNTs(4 wt%)/PMMA, PHB/a-MWCNTs(4 wt%)/PMMA, PHB/f-MWCNTs(4 wt%)/PMMA and PHB/g-MWCNTs(4 wt%)/PMMA (inset) is shown in Figure 12. Figure 12 demonstrates that when compared to all other synthesized nanocomposite sensors, PHB/g-MWCNTs(4 wt%)/PMMA sensors have a 20-fold increase in sensitivity (≈100%). The PHB/g-MWCNTs(4 wt%)/PMMA sensor in the inset of Figure 12 also depicts excellent sensitivity when exposed to low concentrations of NH_3_ as compared to other nanocomposites. All the fivesets of the gas sensors were also examined under continuous exposure of ammonia gas (250 ppm) at room temperature (Figure 13a–d). Only PHB/g-MWCNTs(4 wt%)/PMMA (Figure 13d) displays outstanding repeatability with a steady response up to five cycles. The explanation for this could be related to the porous structure created by wrapping g-MWCNTs in a PMMA/PHB blend. The porous construction allows for easy ammonia gas diffusion, resulting in rapid reaction and good reversibility.

## 4. Conclusions

The surface functionalization of MWCNTs resulted in uniform distribution and robust interfacial interactions with PMMA alone and a PHB/PMMA blend, according to FTIR and SEM studies. The integration of f-MWCNTs and g-MWCNTs in PMMA and PHB/PMMA could significantly improve the energy storage capability, according to a capacitance test conducted on nanocomposites. As a result, the fabricated nanocomposites i.e., f-MWCNTs(4wt%)/PMMA, g-MWCNTs(4 wt%)/PMMA, PHB/f-MWCNTs(4 wt%)/PMMA, PHB/g-MWCNTs(4 wt%)/PMMA are expected to find application as the capacitor materials. In comparison to the neat PMMA dielectric capacitor, the dielectric nanocomposites’ increased capacitance will boost the energy storage capacity. Nanocomposites including functionalized MWCNTs (f-MWCNTs and g-MWCNTs) have better thermal properties than those containing p-MWCNTs, according to the thermogravimetric study. The greater dispersion of MWCNTs is responsible for the increase in the thermal degradation temperature of nanocomposites as compared to neat PMMA and PHB. As a result, functionalization is a necessary step in the reinforcement effectiveness. MWCNTs in nanocomposites act as restriction sites, reducing the segmental mobility of polymeric chains. The lower mobility helps to improve the thermal stability of nanocomposites by delaying chain scission [83]. PHB/f-MWCNTs(4 wt%)/PMMA and PHB/g-MWCNTs(4 wt%)/PMMA nanocomposites have improved thermal stability, making them ideal materials for synthesizing high-performance nanocomposites. Among the nanocomposites PHB/g-MWCNTs(4 wt%)/PMMA showed better ammonia sensing response. The sensor has a high sensitivity for detecting ammonia (97% for 250 ppm).

## Figures and Tables

**Table 1:** TGA and dTGA data of MWCNTs(4 wt%)/PMMA and PHB/MWCNTs/PMMA nanocomposites.

Samples	T_5_/°C	T_10_/°C	T_50_/°C	T_max_/°C	T_end_/°C	Residual Material at 400 °C
PMMA	154	170	317	368	396	1.07%
PHB	-	-	-	-	314	0%
p-MWCNTs(4 wt%)/PMMA	156.8	180.4	339.4	375	395	4.4%
a-MWCNTs(4 wt%)/PMMA	128	163.5	375.7	383	411	15%
f-MWCNTs(4 wt%)/PMMA	123	150.31	378.2	385.4	414	16%
g-MWCNs(4 wt%)/PMMA	123	148.4	378	385	414	17%
PHB/p-MWCNTs(4 wt%)/PMMA	123.5	271.3	376	388.6	425	21%
PHB/a-MWCNTs(4 wt%)/PMMA	113	264	339.4	389	425	22%
PHB/f-MWCNTs(4 wt%)/PMMA	281	296	382.5	393	424	23%
PHB/g-MWCNs(4 wt%)/PMMA	149	287	379	390	424	21%

## Data Availability

Not applicable.

## Supplementary Materials

The following are available online at https://www.mdpi.com/article/10.3390/nano11102625/s1, Test S1, Figure S1: FTIR spectra of neat (a) PMMA and (b–e) MWCNTs(4 wt%)/PMMA nanocomposites, Figure S2: Shifting of peaks in the range (A) 2970–1270 cm^−1^, (B) 1000–800 cm^−1^, and (C) new peaks appearance in FTIR spectra of PMMA and MWCNTs(4-wt%)/PMMA nanocomposites, Figure S3: Spectra of (a) PMMA, (b–e) PHB/MWCNTs(4 wt%)/PMMA/ nanocomposites and (f) PHB, Figure S4: SEM images, Table S1: AC conductance of MWCNTs(4 wt%)/PMMA and PHB/MWCNTs(4 wt%)/PMMA nanocomposites at 25 Hz, Text S2. Scheme S1. A hypothetical model of hydrogen bonding between a-MWCNTs and PMMA. Scheme S2. A hypothetical model of hydrogen bonding between f-MWCNTs and PMMA. Scheme S3. A hypothetical model of hydrogen bonding between g-MWCNTs and PMMA. Scheme S4 (a) and (b): Hypothetical models of hydrogen bonding between f-MWCNTs, PMMA and PHB.
