# Supplementary material for: Effect of MWCNTs Functionalization on Thermal, Electrical, and Ammonia-Sensing Properties of MWCNTs/PMMA and PHB/MWCNTs/PMMA Thin Films Nanocomposites
Source: Nanomaterials (Basel). 2021 Oct 6;11(10):2625. doi: 10.3390/nano11102625 (PMC8539491; doi:10.3390/nano11102625)
Supplement: Supplementary file 1 [file nanomaterials-11-02625-s001.zip › nanomaterials-1361379-supplementary.pdf]

nanomaterials

MDPI

Supplementary Materials

Effect of MWCNTs Functionalization on Thermal, Electrical, and Ammonia-Sensing Properties of MWCNTs/PMMA and PHB/MWCNTs/PMMA Thin Films Nanocomposites

Raina Aman Qazi [1,2,*], Rozina Khattak [2,*], Luqman Ali Shah [1], Rizwan Ullah [1], Muhammad Sufaid Khan [3], Muhammad Sadiq [4], Mahmoud M. Hessien [5] and Zeinhom M. El-Bahy [6]

1. Polymer Laboratory, National Centre of Excellence in Physical Chemistry, University of Peshawar, Peshawar 25120, Pakistan; luqman_alisha@yahoo.com (L.A.S.); drrizwan@uop.edu.pk (R.U.)
2. Department of Chemistry, Shaheed Benazir Bhutto Women University, Peshawar 25000, Pakistan
3. Department of Chemistry, University of Malakand, Chakdara 18800, Pakistan; sufaid1984@uom.edu.pk
4. Department of Chemistry, Quaid-i-Azam University, Islamabad, Pakistan; m_sidiq12@yahoo.com
5. Department of Chemistry, College of Science, Taif University, P.O. Box 11099, Taif 21944, Saudi Arabia; m.hessien@tu.edu.sa
6. Department of Chemistry, Faculty of Science, Al-Azhar University, Nasr City 11884, Cairo, Egypt; zeinelbahy@azhar.edu.eg

* Correspondence: rainaaman@sbbwu.edu.pk (R.Q.); rznkhattak@sbbwu.edu.pk (R.K.)

Text S1:

1.1. FTIR Spectra of MWCNTs/PMMA Nanocomposites

The FTIR spectra of MWCNTs(4 wt%)/PMMA nanocomposites and neat PMMA are presented in Figure S1. Figures S2A–C (extended form of the FTIR results) show that in comparison to pure PMMA, the shift of peaks in MWCNTs(4 wt%)/PMMA nanocomposites was observed at 2952 cm^{-1}, 2850 cm^{-1}, (-CH_3 and –CH_2 bond stretching), 1724 cm^{-1}(C=O), 1261 cm^{-1}(C-C-O), 988 cm^{-1}(O-CH_3 rock or CH bending vibration), 822 $cm^{-1}$and 804 cm^{-1}, which confirms the presence of hydrogen bonds in all synthesized nanocomposites [1]. The peak shift at 2850 and 1724 is only prominent for g-MWCNTs(4 wt%)/PMMA nanocomposite. FTIR result depicts that in hydrogen bonded systems, some shifts occur in a bathocromic way (red shift) and others in a hypsochromic one (blue shift). According to Mitradip et al. the frequency shifts in the X-H-Y hydrogen bonded systems can be correlated with the Mulliken charges on Y and H. The negative charge on Y (electron rich Y) pulls the hydrogen toward itself and causes an elongation of X-H bond, results in red shift of the frequency of X-H vibration. The positive charge on Y shortens the X-H bond length due to electrostatic repulsion of H toward X, resulting in blue shift [2]. The appearance of new peaks in f-MWCNTs(4 wt%)/PMMA (3384 cm^{-1}, 1635 cm^{-1}, 1596 cm^{-1}, 1505 $cm^{-1}$and 693cm^{-1}) and g-MWCNTs(4 wt%)/PMMA (3395 cm^{-1},1645 cm^{-1}, 1596 cm^{-1}, and 693 cm^{-1}) nanocomposites were observed. These peaks are more prominent in f-MWCNTs(4 wt%)/PMMA as compared to g-MWCNTs/PMMA nanocomposites. The a-MWCNTs has C=O and O-H groups which can act as a hydrogen bond acceptor and donors respectively. In f-MWCNTs the hydrogen bond donors are -NH_3 and –NH_2 groups. The hypothetical models of hydrogen bonding between PMMA and a-MWCNTs, f-MWCNTs and g-MWCNTs are shown in schemes 1–3 respectively. From the hypothetical models, it can be seen that the possibility of hydrogen bonding is more prominent in f-MWCNTs/PMMA in contrast to a-MWCNTs(4 wt%)/PMMA and g-MWCNTs(4 wt%)/PMMA. Thus, FTIR studies and hypothetical models are supporting each other.

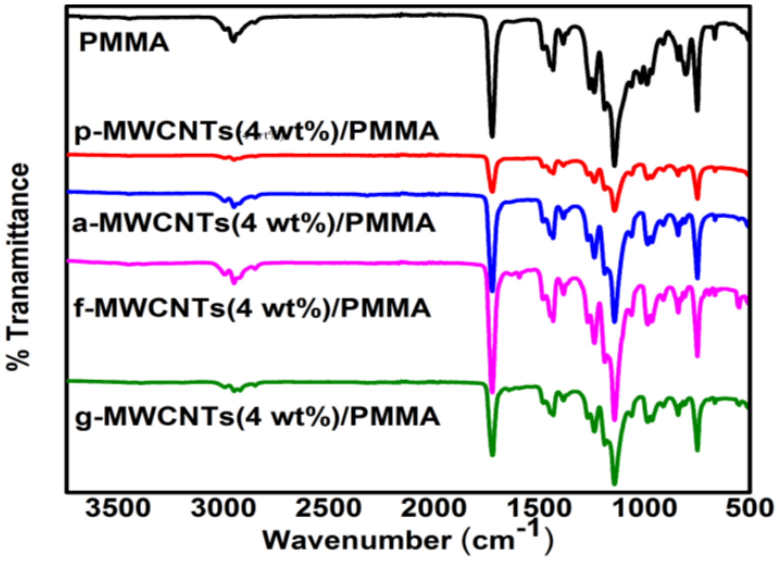

Figure S1. FTIR spectra of neat (**a**) PMMA and (**b**–**e**) MWCNTs (4 wt%)/PMMA nanocomposites.

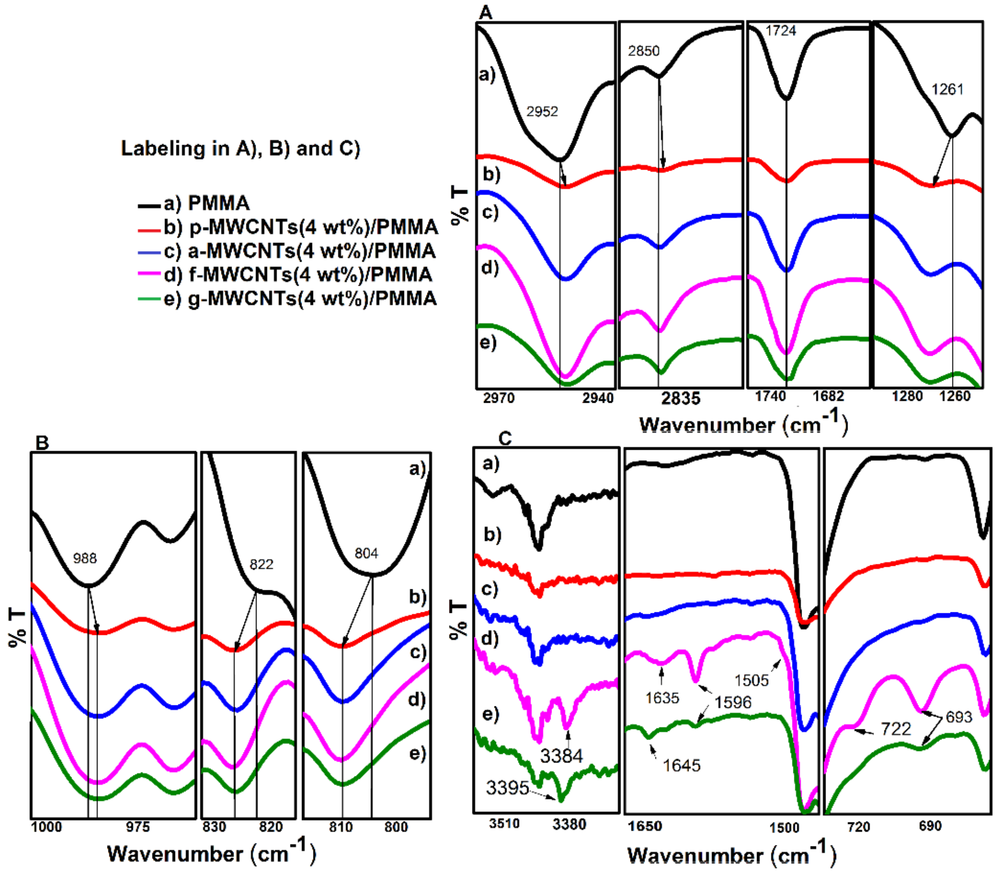

Figure S2. Shifting of peaks in the range (**A**) 2970–1270 cm^{-1}, (**B**) 1000–800 cm^{-1}, and (**C**) appearance of new peaks in FTIR spectra of PMMA and MWCNTs (4-wt%)/PMMA nanocomposites.

1.2. FTIR Spectra of PHB/MWCNTs(4 wt%)/PMMA Nanocomposites

FTIR spectra of neat PMMA, PHB/p-MWCNTs (4 wt%)/PMMA, PHB/a-MWCNTs (4 wt%)/PMMA, PHB/f-MWCNTs (4 wt%)/PMMA and PHB/g-MWCNTs(4 wt%)/PMMA nanocomposites and PHB are presented in Figure S3a–e, respectively. The PMMA peaks (Figure S3a) at 2952 cm^{-1}, 2850 cm^{-1}(-CH_3 and –CH_2 bond stretching), 1724 cm^{-1}(C=O), 1261 cm^{-1}(C-C-O), 988 cm^{-1}(O-CH_3 rock or CH bending vibration) [1], 822 cm^{-1}, 804 cm^{-1}(CH_3 rocking vibrations) [3,4] showed shift in their position in case of all PHB/MWCNTs/PMMA nanocomposites (Figure S3 (b–d)). Thus, confirming the exist-

ence of hydrogen bonds in all synthesized nanocomposites [5,6]. Peak shift at 2850 $cm^{-1}$and 1724 $cm^{-1}$is only prominent for PHB/g-MWCNTs (4 wt%)/PMMA**.** However, the FTIR spectrum of PHB/f-MWCNTs(4 wt%)/PMMA (Figure S3 (c)) depicts new peaks. The appearance of new peaks indicates the intermolecular interaction of f-MWCNTs within PMMA/PHB blend system [7]. The FTIR results verify the existence of well-dispersed f-MWCNTs in PMMA/PHB blend. The existence of strong interaction in PHB/f-MWCNTs(4 wt%)/PMMA composite can be explained due to the fact that $-NH_2$ and –NH groups in f-MWCNTs can form hydrogen bonds more effectively with C=O of both PMMA and PHB. A peak at 1724 cm^{-1}assigned to C=O in neat PMMA (Figure S3 (a)) is shifted to lowest value of 1720 $cm^{-1}$for PHB/f-MWCNTs(4 wt%)/PMMA (Figure S3 (d)) among all synthesized nanocomposites. Schemes 4a,b represent a hypothetical model of hydrogen bonding between f-MWCNTs, PMMA, and PHB.

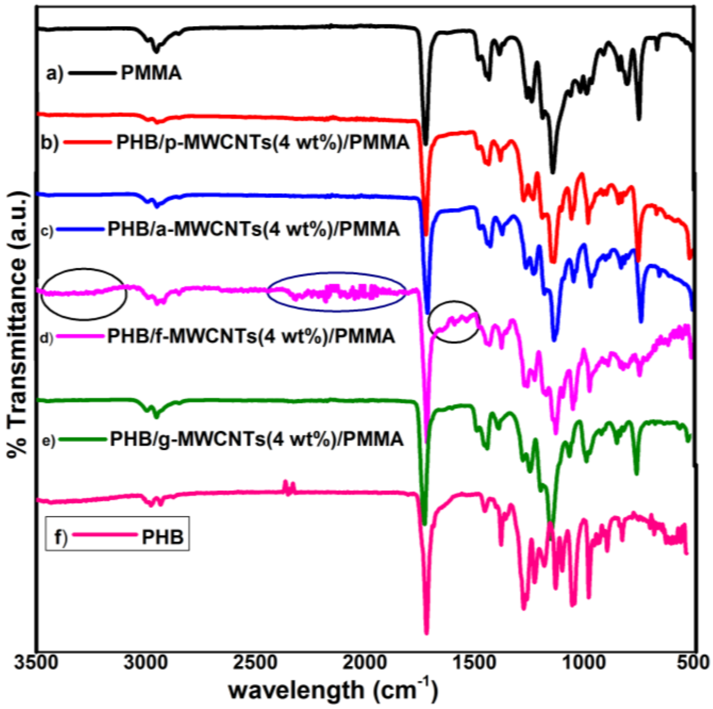

Figure S3. Spectra of (**a**) PMMA, (**b**–**e**) PHB/MWCNTs(4 wt%)/PMMA/ nanocomposites, and (**f**) PHB.

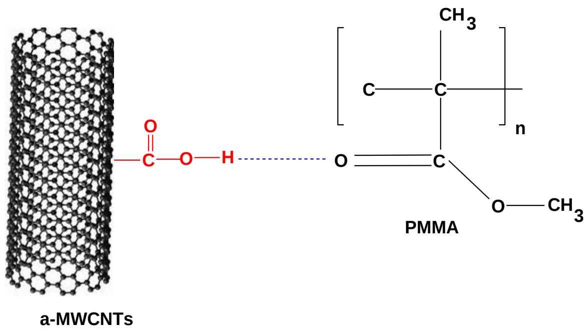

Scheme S1. A hypothetical model of hydrogen bonding between a-MWCNTs, and PMMA.

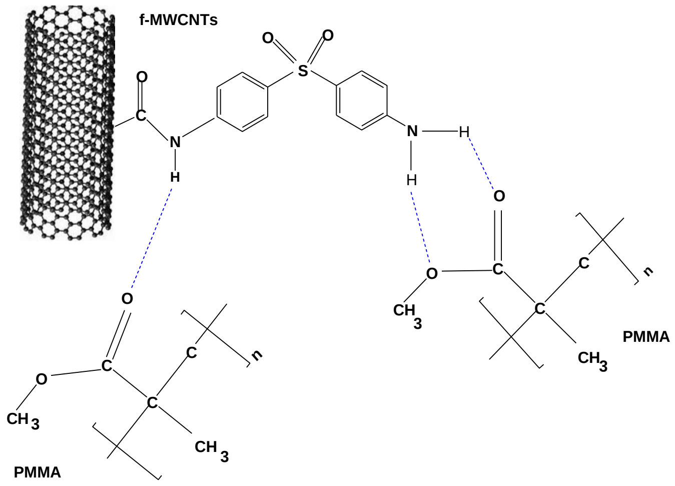

Scheme S2. A hypothetical model of hydrogen bonding between f-MWCNTs, and PMMA.

Text S2

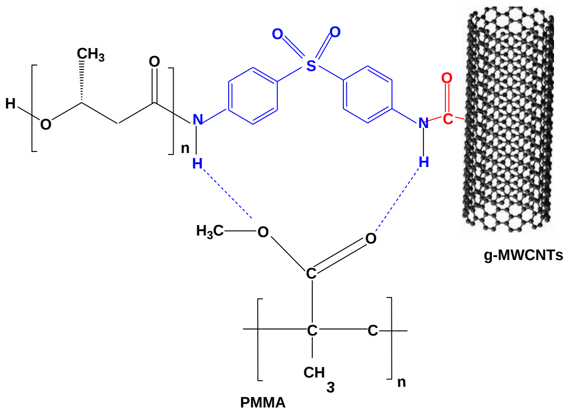

Scheme S3. A hypothetical model of hydrogen bonding between g-MWCNTs, and PMMA.

a)

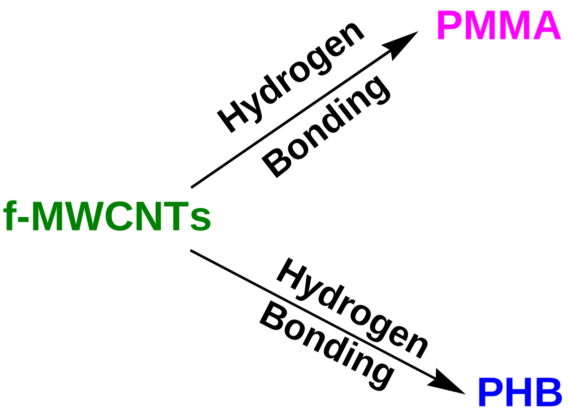

b)

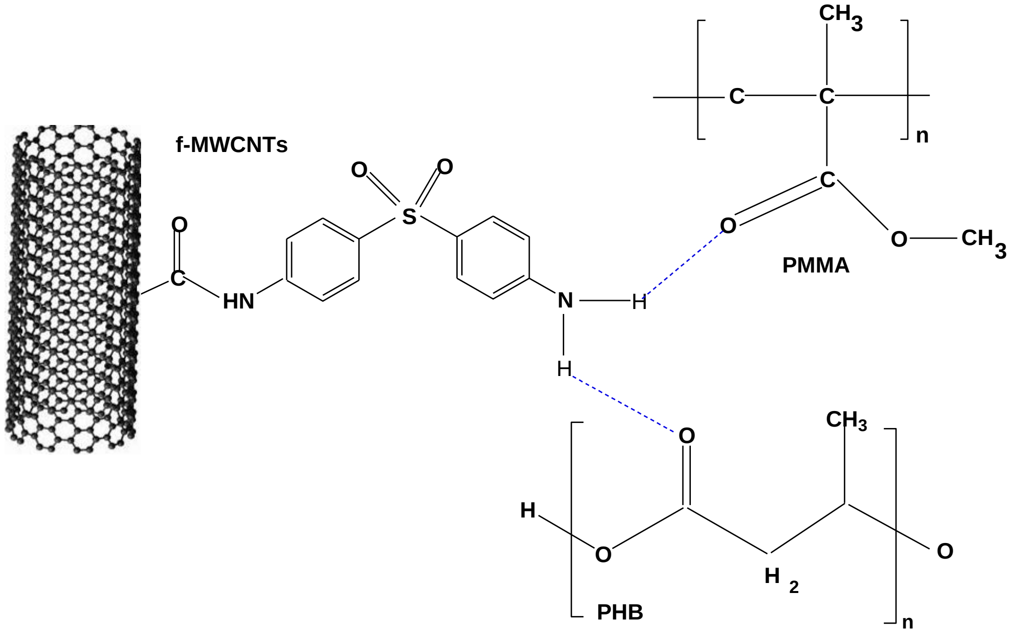

Scheme S4. (**a**) and (**b**). The hypothetical models of hydrogen bonding between f- MWCNTs, PMMA and PHB.

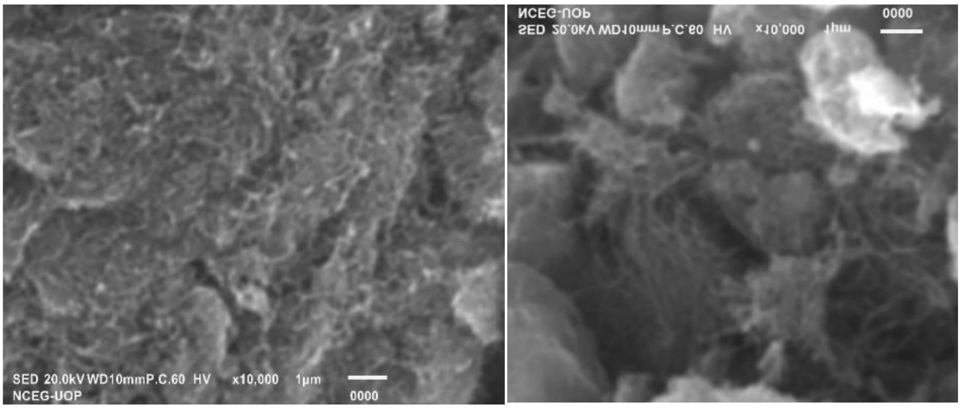

a) p-MWCNTs **b) g-MWCNTs**

Figure S4. SEM images.

Table S1. AC conductance of MWCNTs (4 wt%)/PMMA and PHB/MWCNTs (4 wt%)/PMMA nanocomposites at 25Hz.

Sample	AC Conductance (S)
PMMA	1.7331× 10–10
p-MWCNTs (4wt%)/PMMA	0.1767
a-MWCNTs (4wt%)/PMMA	0.0340
f-MWCNTs (4wt%)/PMMA	0.0117
g-MWCNTs(4wt%)/PMMA	8.26× 10–4
PHB/p-MWCNTs(4wt%)/PMMA	0.01083
PHB/a-MWCNTs(4wt%)/PMMA	0.00187
PHB/f-MWCNTs(4wt%)/PMMA	1.0041× 10–4
PHB/g-MWCNTs(4wt%)/PMMA	4.1825× 10–5

Text S2: The electrical conductance vs. frequency plots shows three types of behavior. Below the percolation threshold, the AC conductance increases linearly with the in-

creasing frequency and the nanocomposites behave as dielectric material where the conductivity can be expressed by the equation (1) [8].

$$\sigma = \omega\varepsilon''\varepsilon^{\circ} \quad (1)$$

Where ω is the angular frequency and ε° is the permittivity of the vacuum. At a percolation threshold, the AC conductivity becomes equal to DC conductivity up to a certain characteristic frequency after which the conductance increases again in the dielectric but with a slightly lower slope. Beyond the percolation threshold, the conductance remains constant with increasing frequency in the given range.

Below percolation threshold, the electron hopping is the main conduction mechanism. Near or at the percolation threshold, AC conduction occurs through electron tunneling and capacitive paths formed among nanoparticles in the polymer matrix. Once the percolation limit is attained, direct contact among the filler particles and electron tunneling becomes the main AC conduction mechanisms in polymer nanocomposites [9]. PMMA exhibits strong frequency dependence, i.e., AC conductivity increases with an increase in frequency, which is characteristic of an electrical insulator [10]. Insulating polymers can be converted to electrically conductive materials by the incorporation of conductive fillers. These fillers will start to create conductive pathways. The concentration at which the randomly dispersed interconnected fillers form percolating networks in the insulating matrix is known as the percolation threshold [11,12].

References

1. Bharti, M. L.; Dutt, S.; Raturi, R.; Joshi, V. Structural Modifications of PMMA and PMMA/CNT Matrix by Swift Heavy Ions Irradiation. In IOP Conference Series. *Materials Science and Engineering* (Online), **2017**, *225*, 012093.
2. Das, M.; Ghosh, S. K. A computational investigation of the red and blue shifts in hydrogen bonded systems. *Journal of Chemical Sciences***2017**, *129*, 975–981.
3. Gavade, C.; Singh, N.; Singh, D.; Shah, S.; Tripathi, A.; Avasthi, D. Study of dielectrical properties of swift heavy ion induced modifications in metal oxide/PMMA nanocomposites. *Integrated Ferroelectrics***2010**, *117*, 76–84.
4. Haris, M.; Kathiresan, S.; Mohan, S. FT-IR and FT-Raman spectra and normal coordinate analysis of poly methyl methacrylate. *Der Pharma Chemica***2010**, *2*, 316–323.
5. Olivieri, G.; Cossaro, A.; Capria, E.; Benevoli, L.; Coreno, M.; De Simone, M.; Prince, K. C.; Kladnik, G.; Cvetko, D.; Fraboni, B. Intermolecular Hydrogen Bonding and Molecular Orbital Distortion in 4-Hydroxycyanobenzene Inves-tigated by X-ray Spectroscopy. *The Journal of Physical Chemistry C***2015**, *119*, 121–129.
6. Mohanraj, J.; Capria, E.; Benevoli, L.; Perucchi, A.; Demitri, N.; Fraleoni-Morgera, A. XRD-and infrared-probed ani-sotropic thermal expansion properties of an organic semiconducting single crystal. *Physical Chemistry Chemical Physics* **2018**, *20*, 1984–1992.
7. Nihmath, A.; Ramesan, M. Fabrication, characterization and dielectric studies of NBR/hydroxyapatite nanocompo-sites. *Journal of Inorganic and Organometallic Polymers and Materials***2017**, *27*, 481–489.
8. Kim, Y. J.; Shin, T. S.; Do Choi, H.; Kwon, J. H.; Chung, Y.-C.; Yoon, H. G. Electrical conductivity of chemically modi-fied multiwalled carbon nanotube/epoxy composites. Carbon 2005, 43, 23–30.
9. Chang, J.; Liang, G.; Gu, A.; Cai, S.; Yuan, L. The production of carbon nanotube/epoxy composites with a very high dielectric constant and low dielectric loss by microwave curing. *Carbon***2012**, *50*, 689–698.
10. Thakre, P. R.; Bisrat, Y.; Lagoudas, D. C. Electrical and mechanical properties of carbon nanotube ‐ epoxy nanocom-posites. *Journal of applied polymer science***2010**, *116*, 191 ‐ 202.
11. Weber, M.; Kamal, M. R. Estimation of the volume resistivity of electrically conductive composites. *Polymer compo-sites***1997**, *18*, 711–725.
12. Sandler, J.; Shaffer, M.; Prasse, T.; Bauhofer, W.; Schulte, K.; Windle, A. Development of a dispersion process for carbon nanotubes in an epoxy matrix and the resulting electrical properties. *Polymer***1999**, *40*, 5967–5971.
